# Brain Connectivity and Information-Flow Breakdown Revealed by a Minimum Spanning Tree-Based Analysis of MRI Data in Behavioral Variant Frontotemporal Dementia

**DOI:** 10.3389/fnins.2019.00211

**Published:** 2019-03-14

**Authors:** Valentina Saba, Enrico Premi, Viviana Cristillo, Stefano Gazzina, Fernando Palluzzi, Orazio Zanetti, Roberto Gasparotti, Alessandro Padovani, Barbara Borroni, Mario Grassi

**Affiliations:** ^1^Medical and Genomic Statistics Unit, Department of Brain and Behavioral Sciences, University of Pavia, Pavia, Italy; ^2^Neurology Unit, Department of Clinical and Experimental Sciences, Centre for Neurodegenerative Disorders, University of Brescia, Brescia, Italy; ^3^Alzheimer’s Research Unit, IRCCS Fatebenefratelli, Brescia, Italy; ^4^Neuroradiology Unit, University of Brescia, Brescia, Italy

**Keywords:** functional connectivity, functional magnetic resonance imaging, resting state, minimum spanning tree, graph theory, behavioral variant frontotemporal dementia, neurodegeneration

## Abstract

Brain functional disruption and cognitive shortfalls as consequences of neurodegeneration are among the most investigated aspects in current clinical research. Traditionally, specific anatomical and behavioral traits have been associated with neurodegeneration, thus directly translatable in clinical terms. However, these qualitative traits, do not account for the extensive information flow breakdown within the functional brain network that deeply affect cognitive skills. Behavioural variant Frontotemporal Dementia (bvFTD) is a neurodegenerative disorder characterized by behavioral and executive functions disturbances. Deviations from the physiological cognitive functioning can be accurately inferred and modeled from functional connectivity alterations. Although the need for unbiased metrics is still an open issue in imaging studies, the graph-theory approach applied to neuroimaging techniques is becoming popular in the study of brain dysfunction. In this work, we assessed the global connectivity and topological alterations among brain regions in bvFTD patients using a minimum spanning tree (MST) based analysis of resting state functional MRI (rs-fMRI) data. Whilst several graph theoretical methods require arbitrary criteria (including the choice of network construction thresholds and weight normalization methods), MST is an unambiguous modeling solution, ensuring accuracy, robustness, and reproducibility. MST networks of 116 regions of interest (ROIs) were built on wavelet correlation matrices, extracted from 41 bvFTD patients and 39 healthy controls (HC). We observed a global fragmentation of the functional network backbone with severe disruption of information-flow highways. Frontotemporal areas were less compact, more isolated, and concentrated in less integrated structures, respect to healthy subjects. Our results reflected such complex breakdown of the frontal and temporal areas at both intra-regional and long-range connections. Our findings highlighted that MST, in conjunction with rs-fMRI data, was an effective method for quantifying and detecting functional brain network impairments, leading to characteristic bvFTD cognitive, social, and executive functions disorders.

## Introduction

The bvFTD is clinically defined by personality changes and behavioral disturbances, impairment of executive functions and emotional blunting (Gorno-Tempini et al., 2011; Rascovsky et al., 2011). Abnormal intracellular accumulation of either tau or TDP-43 protein is found in most cases (Mann and Snowden, 2017).

Recently, the increasing interest in unraveling functional and structural features of the brain, has benefitted from complex network analyses, such as graph theory, a multidisciplinary approach that allows to analyse complex systems in a straightforward computable way and to describe cerebral areas as nodes, and their connections as edges (Rubinov and Sporns, 2010). Both structural (anatomical) and functional (statistical relationship between two nodes) connectivity can be assessed (Zhang et al., 2016).

Applying graph theoretical methods to neuroimaging techniques is becoming popular in the study of brain dysfunction (Bullmore and Sporns, 2009; Drakesmith et al., 2015; Chiang et al., 2016). Recently, a few studies have considered graph theory analysis applied to rs-fMRI data in patients with bvFTD and have better described FTD-related brain changes (Agosta et al., 2013; Filippi et al., 2017). However, although conventional graph theoretical analyses are helpful in dissecting disease mechanisms (Bullmore and Sporns, 2012), the methodology is significantly hampered by a number of arbitrary choices. Descriptive metrics and their normalization, network type (weighted or unweighted networks), threshold value (fixed cut-off, fixed average degree, fixed edge density, or variable threshold over a range of values) are some of the critical points making network results difficult to reproduce (van Wijk et al., 2010; Telesford et al., 2011; Stam et al., 2014; Drakesmith et al., 2015; Yu et al., 2016). In addition, several network metrics and node centrality indices may assume different importance at either local or global scale (Telesford et al., 2011; Antonenko et al., 2018), whether the graph model accounts for time variant (i.e., dynamic) or invariant (i.e., static) connectivity (Rashid et al., 2014; Park et al., 2018), and the parcellation type, according to Independent Component Analysis (ICA; McKeown et al., 2003; Griffanti et al., 2014) and specific atlases (see Materials and Methods, for data pre-processing in this work).

Minimum Spanning Tree, a unique acyclic subgraph that connects *N* nodes with (*N*-1) edges, and maximizing synchronization between brain areas (i.e., minimizes edge connections), is a promising unambiguous solution to describe complex brain networks (Stam et al., 2014). The use of MST avoids methodological confounding thanks to an efficient integration of topological properties and functional connectivity information (Tewarie et al., 2014; van Diessen et al., 2016; van Lutterveld et al., 2017), ensuring network robustness and reproducibility respect to classical graph analytical approaches (Otte et al., 2015; Tewarie et al., 2015). MST is a tree which has the minimum total edge weight of all possible spanning trees of the original graph. If the brain network can be interpreted as a kind of transport network, an MST might represent the critical backbone of information flow in weighted networks (i.e., contains with high probability all the shortest paths in the network). Given MSTs efficiency and high sensitivity to small fluctuations of connection weights (Van Mieghem and Magdalena, 2005), they can intrinsically provide an accurate representation of subtle and critical topological perturbations, at local scale. On the other hand, MST sparseness could raise issues at global scale. However, it has been demonstrated that if the MST weight distribution is consistent with a power law with sufficiently small exponent value, the global information flow of the underlying network follows entirely MST paths (Van Mieghem and Magdalena, 2005; Van Mieghem and van Langen, 2005; Meier et al., 2015). Moreover, TOMs can be applied to the original adjacency matrix to modulate neighborhood characteristics in MST nodes (Ravasz et al., 2002). In addition, we used wavelet decomposition and correlation to obtain noise-free and robust functional relationships between brain areas (Achard et al., 2006; Zhang et al., 2016).

Collectively, these aspects enable data-driven network comparison of healthy and diseased groups, without normalization or standardization steps, as recently illustrated in EEG and MEG data (Stam et al., 2014; van Dellen et al., 2014; Numan et al., 2017). Furthermore, network robustness and reproducibility should ensure univocal results, minimizing room for ambiguous interpretations. Besides graph theoretical aspects, data acquisition and pre-processing issues may affect results, including brain parcellation and data acquisition technologies. These aspects should follow the principles of common usage, availability, cost effectiveness, and non-invasivity, that can secondarily affect methodological choices and issues (Hohenfeld et al., 2018). Despite objective difficulties in generating a consensus functional brain map, especially for rare disorders, a set of reference resting state functional networks have been replicated in many different studies (Hohenfeld et al., 2018). According to well-established rs-fMRI literature, three reference networks are mainly involved in bvFTD functional breakdown: the default-mode network (DMN), the salience network (SN), and the executive network (EN), accounting for the cognitive, emotional, and social impairments characterizing this pathology (Raichle, 2015; Trojsi et al., 2015; Sedeño et al., 2016; Hohenfeld et al., 2018).

In this study, we leverage MST-based analysis of rs-fMRI data to investigate large-scale functional network alterations, inspecting global and local network properties in bvFTD patients, compared to a group of HC, and providing a novel MST procedure, combining individual tree-based global evidences and two-group topological aspects.

## Materials and Methods

**FIGURE 1:**
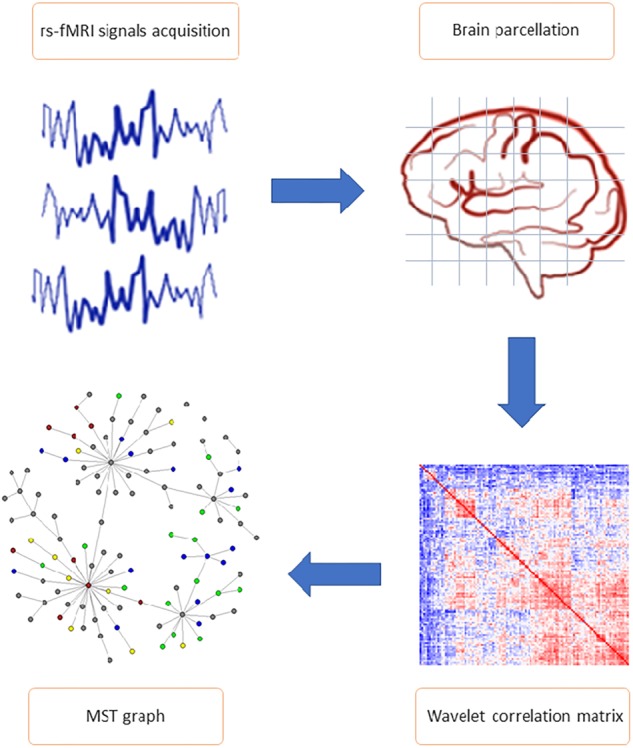
General study workflow. The most important steps of connectome extraction. MRI signals acquisition and brain parcellation represent the first phases of resting state functional magnetic resonance imaging data pre-processing. Wavelet correlation matrix and minimum spanning tree (MST) network calculation are included in the analysis phase. Wavelet transformation was applied to the average voxel time series mapped to AAL 116 brain regions of interest (ROI), allowing to obtain the statistical relationships between nodes (i.e., wavelet correlation matrices). Each matrix is the input of the MST algorithm, from which parameters and topological features (including edges partition and nodes cluster) have been calculated.

The analysis workflow of the rs-fMRI data is illustrated in Figure 1 and described in detail below.

### Subjects

Forty-one patients with a probable bvFTD diagnosis, according to current criteria (Gorno-Tempini et al., 2011), were recruited at the Center for Neurodegenerative Disorders, University of Brescia, Italy All patients underwent an extensive neuropsychological assessment, as previously published (Gazzina et al., 2016), genetic screening for the most frequent monogenic causes of FTD (i.e., *Granulin, C9orf72*, and *Microtubule Associated Protein* Tau; Cosseddu et al., 2018) and brain MRI structural imaging study. In the present study, none of the bvFTD cases carried pathogenic mutations of monogenic bvFTD.

Thirty-nine HC, recruited from voluntary individuals, were used as control group. HC underwent a brief standardized neuropsychological assessment (Mini-Mental State Examination; MMSE >=27). Table 1 shows the demographic information of the participants. The study, in conformity with the Helsinki Declaration, was approved by the Brescia Hospital Ethics Committee. Informed consent was obtained from all participants.

**Table 1:** Demographic and clinical characteristic of the participants.

	bvFTD (*n* = 41)	HC (*n* = 39)	*P*-value
Age (year)	65.6 ± 7.01	61.7 ± 6.5	0.010*^a^*
Sex (male/female)	26/15	13/26	0.014*^b^*
Education (year)	9.1 ± 3.82	10.3 ± 3.84	0.161*^a^*
CDR	6.7 ± 3.84	0.0 ± 0.0	–

*Values for bvFTD and HC are reported as mean ± SD; *a*, independent two-group *t*-test; *b*, Pearson chi-squared test; CDR, Clinical Dementia Rating.*

### MRI Acquisition

All imaging was obtained using a 1.5T Siemens Avanto MRI scanner (Siemens, Erlangen, Germany), equipped with a circularly polarized transmit-receive coil. In a single session, the following scans were collected from each subject:

(i) dual-echo TSE [repetition time = 2500 ms, echo time (TE) = 50 ms], to exclude presence of macroscopic brain abnormalities, according to exclusion criteria;
(ii) 3D MPRAGE T1-weighted scan (TR = 2050 ms, TE = 2.56 ms, matrix = 1 × 1 × 1, in-plane field of view [FOV] = 256 × 256 mm^2^, slice thickness = 1 mm, flip angle = 15°);
(iii) T2^∗^-weighted EPI sensitized to BOLD contrast [TR = 2500 ms, TE = 50 ms, 29 axial slices parallel to anterior commissure-posterior commissure line (AC-PC) line, matrix = 64 × 64, field of view = 224 mm, slice thickness = 3.5 mm], gap between slices 1.75 mm for rs-fMRI.

Echo planar images were collected during rest for an 8-min period, resulting in a total of 195 volumes.

### Neuroimaging Pre-processing

Functional data were pre-processed using FSL 5.0.8 neuroimaging software, as reported in Jenkinson et al. (2012): (i) the first two volumes were removed to allow signal stabilization; each volume was motion-corrected to a reference volume using MCFLIRT; (ii) non-brain structures were removed using Brain Extraction Tool (BET); (iii) the effect of TR during slice acquisition was reduced using slice-timing correction and the data were spatially smoothed applying the Gaussian kernel with a full width and half maximum (FWHM) of about 7 mm; (iv) grand-mean intensity of the entire data was adjusted by a single multiplicative factor; (v) high-pass temporal filtering Gaussian-weighted least-squares straight line fitting (100 s) was applied; (vi) functional data were co-registered into equivalent native-space T1 weighted image using Boundary-Based Registration (BBR); and (vii) each T1 weighted image was co-registered into standard space template MNI152 using linear (affine with 12 degree of freedom) brain image registration (FLIRT).

To control for motion effect, we included in the further steps only subjects with head motion in a range below or equal to 1 mm (translation) and one degree (rotation). Motion parameters were derived from six degree of freedom registration using MCFLIRT (Jenkinson et al., 2012).

After pre-processing, we applied an automatic approach called “FMRIB’s ICA-based X-noiseifier” (FIX) to detect non-signal components in resting state images which combines the classifiers approach and the Independent Component Analysis (ICA) in a MATLAB environment (Griffanti et al., 2014, 2015). We applied FIX procedure in three steps. First, for each subject we estimate the amount of Gaussian noise of the true dimensionality of the data, i.e., the number of activation and non-Gaussian noise sources using a probabilistic ICA approach implemented in MELODIC (Beckmann and Smith, 2004). Then, we made a random selection of subjects (10 healthy subjects and 10 bvFTD subjects, covering approximately 25% of the whole sample set) to create a subsample to train the FIX’s multi-level classifier. For these subjects, we manually selected the components (white matter, susceptibility artifact, head motion, cardiac pulsation) looking into the thresholded spatial map estimated from single-ICA and the power spectrum of the time series for each component (Griffanti et al., 2014). Lastly, to test if FIX successfully detected the noise components and regressed out the variance (including the six motion parameters derived from MCFLIRT), we looked into a sample of subjects to confirm classification of bad components. The “cleaned” rs-fMRI images (after noise and motion variance regression) of each subject were subsequently used for brain parcellation.

### Brain Parcellation

The ALL atlas (AAL; Tzourio-Mazoyer et al., 2002) was used to parcel brain into 116 (90 cortical and subcortical, and 26 cerebellar) regions of interest (ROIs, Supplementary Table 1). Mean time series were extracted from each ROI by averaging the signal from all voxels within each region, using Marsbar software (^1^Brett et al., 2002). A subsequent descriptive aggregation was applied to obtain 8 (right and left) macro-ROI (referred to as lobes or “macro-regions”): Frontal, Insular, Limbic, Occipital, Parietal, Subcortical Gray Matter (SCGM; including Thalamus), Temporal, and Cerebellum (including Vermis).

### Wavelet Correlation Analysis

For each subject, the final dataset (Saba et al., 2018) was composed by 193 mean time-series extracted from each brain region. Different correlation estimates define statistical relationship between brain region pairs (Zalesky et al., 2012): in the present study, we used wavelet correlation.

Each temporal series was decomposed using wavelet analysis and characterized by weighted coefficients, proportional to the total amount of energy emitted from the system, relative to a specific scale and brain location. Considering total energy as a frequency-time, wavelet decomposition enables data processing at different hierarchical scale resolutions. Indeed, low-frequency components correspond to coefficients of approximative scale, while high-frequency components correspond to finer scale coefficients (Bullmore et al., 2004). The high flexibility for non-stationary characteristics of the data in each decomposition scale favors wavelet multi-modularity application to fMRI data. Therefore, wavelet correlation results in a higher robustness and noise reduction, with a more homogeneous representation of the original time series and their transformations (Bullmore et al., 2004; Zhang et al., 2016).

Consistent with (Zhang et al., 2016) guidelines, the maximal overlap discrete wavelet transform (MODWT; Achard et al., 2006) with a Daubechies wavelet filter (length equal to 8), was used to band-pass filter on mean time series, and extract wavelet coefficients for the wavelet scales. Given our (TR = 2500 ms, Nyquist frequency = 0.2 Hz), four frequency bands (i.e., scales) were used; scale one: 0.2–0.1 Hz; scale two: 0.05–0.1 Hz; scale three: 0.025–0.05 Hz; and scale four: 0.013–0.025 Hz. Then, we defined a correlation matrix whose *ij*th elements were set by the estimated wavelet correlations between brain regions *i*$\left(\lambda_{i}^{\left(s\right)}\right)$ and region *j*$\left(\lambda_{j}^{\left(s\right)}\right)$:

(1) $$\begin{array}{c} r_{\mathrm{ij}}=\frac{cov\left(\lambda_{i}^{\left(s\right)};\lambda_{j}^{\left(s\right)}\right)}{\sqrt{var\left(\lambda_{i}^{\left(s\right)}\right)var\left(\lambda_{j}^{\left(s\right)}\right)}} \end{array}$$

we focused on wavelet decomposition scale two (*s* = 2), as only this scale reached the significance in three distinct bvFTD/HC connectivity-based Wilcoxon rank sum tests (Supplementary Table 2). Specifically, we quantified bivariate connectivity of the wavelet correlation matrix for each subject (Lynall et al., 2010), with three global measures: (i) *strength*, defined as the average of columns mean; (ii) *diversity*, defined as the average of the columns variance, and (iii) *zero correlation*, defined as the number of correlations with *P* > 0.05, testing the null hypothesis, H_0_ : ρij = 0.

### Minimum Spanning Tree (MST) Structures and Graph Metrics

The MST method overcomes issues concerning arbitrary threshold selection in weighted connected graphs, by joining edges with minimum weight. In other words, it assembles connections minimizing the sum of edge-weights, excluding edges that form a cycle. MST is an extremely efficient binary representation of a full graph *G* (*N, E*) characterized by *N* nodes and *E* = (*N*-1) edges. It yields perfectly comparable networks among different samples, without dependences from vertices and edges number variability, and a best possible synthesis of the original graph information, achieved through the most important subgraph (Stam et al., 2014). Among existing methods for MST search, we applied the Prim’s algorithm (Cormen et al., 2001), to obtain a MST from each subjects’ wavelet correlation matrix.

The MST method handles very different network configurations with their extremes represented by linear or star shapes. In the former, each node has a maximum of two edges (i.e., path-like tree) and two extreme leaves (i.e., nodes with only one link). Whereas, in a star, all nodes are leaves, except the central node to which the other ones are connected (Stam et al., 2014).

Every network can be described through a set of graph metrics (e.g., topological indices) characterized according to its configuration (either linear or star) and its specific graph metrics values. We used global MST measures providing information on graph centrality (maximum degree *k_max_*, maximum betweenness *B*_max_), distance (diameter *d*, eccentricity *Ecc*), association (assortativity *Ass*), and topological aspects (degree divergence *K*, leaf fraction *Lf)*. Their definitions are given in Table 2.

**Table 2:** Description of minimum spanning tree global measures.

Measure	Definition
*k_max_*	*Degree (k)* of a node is the number of links connecting one node to another in the network, and *maximum degree k*_max_ indicates the highest value of node degree in the network
*B*_max_	*Betweenness centrality* (*B)* of a node indicates the number of shortest paths passing through the normalized node such that *B*𝜖[0,1], and *maximum betweenness B*_max_ indicates the highest value of betweenness centrality in the network
*d*	*Diameter (d)* identifies the longest distance, measured in number of edges, between any two nodes; *d* has a range from 2 to *N*-1
*Ecc*	*Eccentricity (Ecc)* of a node indicates the distance of a node with respect to any other node, in term of longest distance between them. Here, Ecc represents the average value (i.e., the arithmetic mean) for all nodes
*Ass*	*Assortativity (Ass)* describes node tendency to link to other nodes, characterized by the same or similar degree, which can be quantified by computing the Pearson correlation coefficient of the degrees of pairs of nodes connected by an edge
*K*	*Degree divergence (K)* is a measure of broadness of the degree distribution in the whole network, defined as the ratio between the variability and the average of node connections
*Lf*	*Leaf fraction (Lf)* is the ratio between the number of nodes with only one edge (i.e., “end-points” in the graph) and the maximum possible number of edges (i.e., the number of nodes minus 1, *N*-1). Stars-type graph is defined by *Lf* = (*N*-1)/(*N*-1) = 1, while linear-type graph by *Lf* = 2/(*N*-1)

*N, number of nodes in a minimum spanning tree.*

Short distances and overload prevention aspects suggest a good tree configuration or network integration. If a tree has a star-like (i.e., highly connected) topology, it will be characterized by a greater information exchange capacity (i.e., spread of information across the tree), yet a greater probability of central node overload. The opposite behavior is true if the graph has a line-like topology. In particular, an increase of maximum betweenness *B*_max_ and leaf fraction *Lf*, with a decrease of diameter d and eccentricity *Ecc*, tend to have a star-type configuration and a better network integration (van Lutterveld et al., 2017). In addition, maximum betweenness *B*_max_ and degree divergence *K* correlate positively with the presence of some high-degree tree nodes (hub communication) in networks with a scale-free degree distribution (a scale-free network has a large number of nodes with a lower degree and few highly connected hubs; Albert and Barabási, 2002; Mears and Pollard, 2016).

Finally, positive assortativity (*Ass* > 0) indicates that nodes are likely to be connected to other nodes with the same degree, and therefore that the high degree nodes (hubs) tend to be connected to each other (Bullmore and Sporns, 2009). Negative assortativity (*Ass <* 0) is typical of biological networks with hierarchical structure where hubs are connected to nodes with lower degree nodes (Newman, 2003).

### Topological Overlap Measures (TOM) and Shortest Path Tree (SPT)

In the present study, we implemented pre-processing procedures to avoid some of the intrinsic limitations of the MST approach: the absence of triangular connections (i.e., absence of clustering metric) e graph sparseness with a limited number of edges of the resulted network.

First, we applied *topological overlap measures (TOM*; Ravasz et al., 2002; Zhang and Horvath, 2005) to the network adjacency matrix to compensate the absence of triangular connections in the MST. This allowed to analyse MSTs in terms of aggregation or clustering without biases. Specifically:

(2) $$\begin{array}{c} TOM_{\mathrm{ij}}=\;\frac{l_{\mathrm{ij}}+\;a_{\mathrm{ij}}\;}{min\left\{k_{i},\;k_{j}\right\}\;+\;1\;-a_{\mathrm{ij}}\;\;} \end{array}$$

where l_ij_ = ∑ _u_a_iu_a_ju_ is the overlap estimate between two nodes neighborhoods, a_ij_ is the ijth-element of adjacency matrix, k_i_ and k_j_ are the degree measures of ith-node and jth-node, expressed as ki = ∑ _u≠i_a_iu_; k_j_ = ∑ _u≠j_a_ju_. A higher overlap is associated with a greater relationship between two nodes, and a greater relationship with their common nodes (Mumford et al., 2010). Therefore, TOM modulates neighborhood characteristics of nodes, by quantifying the topological overlap between two nodes against all other nodes in the network. High TOM values identify nodes that constitute a neighborhood (Ravasz et al., 2002).

Secondly, MST is characterized by (*N*-1) edges, resulting a sparse graph with a limited number of edges. Therefore, we evaluate the network performance of MST through the SPT problem (Van Mieghem and Magdalena, 2005; Meier et al., 2015), quantifying if MST is a good representation of the whole graph (i.e., whether the sparse tree may be considered as a critical backbone of original network; Tewarie et al., 2014).

The shortest path is the path with minimum sum of weights from a source to a destination node, and a SPT is the union of the shortest paths from a source node to all other nodes in the graph. An SPT is mainly sensitive to the small, non-negative link weights, around zero. Starting with a full graph *G*, the probability distribution for the link weights of G around zero can be described by a power distribution: F(x) = Pr (X ≤ x) ~ x^α^, where x ∈ [0,1] represents weights, and the exponent α > 0, defines the *extreme value index* of the probability distribution (Van Mieghem and Magdalena, 2005).

Three specials α-trees correspond to precise α ranges. The α →∞ regime matches a unique weight for all links (i.e., *w* = 1). In the α = 1 regime, link weights result to be uniformly distributed. Finally, in the α → 0 regime, link weights show strong fluctuations. For α → 0, defined as the *stronger disorder condition*, the SPT of the full graph coincide with a MST (Van Mieghem and Magdalena, 2005; Van Mieghem and van Langen, 2005), and the information flow within the network follows only MST links (Meier et al., 2015).

Therefore, we tested the α → 0 regime (i.e., MST) of the weighted full (i.e., original) graph *G* for each subject in three steps:

(1) Define a full graph *G* (*N, E*) by fixing to zero the not statistically significant (*P* > 0.05) wavelet correlations, r_ij_. The null hypothesis: H_0_ : ρij = 0 was evaluated with the Mutual Information test, $MI=-N\;\ln\left(1-r_{\mathrm{ij}}^{2}\right)/2$ (Edwards et al., 2010).
(2) Apply topological overlap measures (i.e., TOM) to the $r_{\mathrm{ij}}^{\left(0\right)}$ = r_ij_ if *P* < 0.05 and $r_{\mathrm{ij}}^{\left(0\right)}$ = 0 if *P* ≥ 0.05. MST was created by edges weights defined as w_ij_ = min(1/*TOM*_ij_; 100)/100. This transformation ensures that weights are enclosed in the range (0, 1), and the most important edges (with small weights, i.e., large TOM values) represent the strongest neighborhood connections.
(3) Ranking weights w_ij_ in descending order and estimating the α exponent from the power functionF (x) = c ⋅ x^α^, by plotting *Y* = log10 [*F*(*x*)] versus *X* = log10 [(x)]. A straight line with an R-squared index approaching 1 is indicative of good fit to the power function, and slope indicates SPT coincidence with MST.

### Edges Partition and Nodes Clustering in MST

Two-group comparisons of topological MST properties were applied to the individual wavelet correlation matrices, between bvFTD and HC, averaged over subjects. Topological structure of nodes and links in an MST have a key role to capture paths with higher importance in information flow (Meier et al., 2015). It is possible to identify an MST subnetwork showing a higher average node (or link) betweenness centrality compared to the rest of MST (Wu et al., 2006). In other terms, this subset of nodes (or links) is used more often than others, and their paths can be considered as a set of *superhighways* (SHW) in MST, i.e., the most important “roads.” Other links in MST constitute “secondary roads.” Identifying SHW enabled to subdivide MST edges (links) in two distinct components with significantly different transport properties.

Based on SHW’s definition, we applied the method suggested by Wu et al. (2006), on both scale-free and Erdos–Renyi (i.e., random) networks, for edges partition in MST. Briefly, considering the fully connected network with the previous TOM-based link weights, we extracted one MST for each case-control group. Through an iterative process, we removed links in descending order of their weights and calculated the degree divergence value (K), which decreases in each cycle with link removals. As demonstrated by Braunstein et al. (2007), the process ends when *K* < 2, and the largest remaining component is the SHW set. Lastly, we measured node betweenness differences (i.e., bvFTD – HC) to assess the amplitude of information flow connectivity gain or loss, and we defined a threshold *b* as the non-zero median of the absolute betweenness differences. Areas showing differences above *b* (or below -*b*), will be taken as markers of functional connectivity gain (or loss).

Clustering nodes in a tree is more complex respect to other graphs. Several types of clustering algorithms have been developed which revealed to be limited and unsuitable to MST characteristics (Yu et al., 2015). Here, we used a novel approach for MST clustering suggested by Yu et al. (2015) based on the geodesic distance matrix D, where the ijth-elements of D represent the number of links of a shortest path between two nodes. As proposed by the authors, we computed vector similarities as the Spearman’s distance, d_S_ = 1 - r_S_, where r_S_ is the Spearman’s rank correlation between all row pairs of D. Next, we applied the iterative hierarchical clustering algorithm, using d_S_ as input and *average-linkage* method (Gordon et al., 2016) to define the distance between two clusters. This method merges node pairs into corresponding clusters by decreasing similarity until all nodes are merged into one cluster. The different stages of the algorithm were represented in the form of a dendrogram. We partitioned the case-control MSTs in an equal number of clusters using the same cut-off value (0.2) on each case-control dendrogram.

### Statistical Analysis

Global network connectivity (strength, diversity, and zero correlation), and MST global parameters (maximum degree, maximum betweenness, diameter, eccentricity, assortativity, degree divergence, leaf fraction, extreme value index, R-squared), provided a dataset of 80 rows (subjects) and 12 columns (parameters) for further statistical analysis. Since data were generally not Gaussian, non-parametric, Wilcoxon signed rank test was used to compare FTD and HC groups *P*-values were adjusted using Benjamini–Hochberg correction, and fixing the significance threshold at *P* < 0.05 (two sided). Sex and age showed significant differences between groups, therefore data were corrected for age, sex and age^∗^sex interaction.

Global measures from the two case-control average correlation matrices were also compared by permutation tests as follows: (i) by computing the observed absolute difference between the global measure in FTD and HC groups, (ii) by permuting group assignments of the individuals’ values of the global measures for FTD and HC groups (*B* = 10000 iterations), and (iii) by repeating step (i) to obtain *B* = 10000 sampled permutations of the absolute differences between FTD and HC groups. Then, *P*-values were obtained using the sample permutation distribution with the same significant threshold of the individual signed rank tests.

### Methodological Comparison

Minimum spanning tree properties were compared with other conventional graph theory approaches, to measure the extent of network metrics reproducibility and wavelet scale specificity. The first method we applied uses the efficiency cost optimization (ECO; De Vico Fallani et al., 2017) criterion, imposing a fixed edge density threshold, based on the trade-off between network efficiency and wiring cost. We further applied two methods defining per subject optimal correlation thresholds, based on the extended Bayesian information criterion (EBIC; Chen and Chen, 2008), and spectral analysis (Perkins and Langston, 2009). Finally, we tested the performances of a scale free model-based method that chooses the correlation threshold optimizing power law fitting for each subject (Mumford et al., 2010). Differently from MST topology, these networks include triangular connections and cycles (i.e., they are not acyclic graphs), where classical clustering-based indices can be calculated. MST-specific indices are leaf fraction and alpha, while non-MST indices include clustering coefficient, average path length, and efficiency. Common metrics include maximum degree, maximum betweenness, degree divergence, diameter, eccentricity, and assortativity. Wilcoxon rank sum tests were calculated for every method at each wavelet scale.

### Software

Network analyses, graph visualization, and statistical analyses were performed in R (R Core team, 2018), using packages *igraph* (Csardi and Nepusz, 2006), *WGCNA* (Langfelder and Horvath, 2008), *brainwaver* (Achard, 2015), *brainGraph* (Watson, 2018), and custom R functions.

## Results

### Global Functional Connectivity of rs-fMRI Data

**FIGURE 2:**
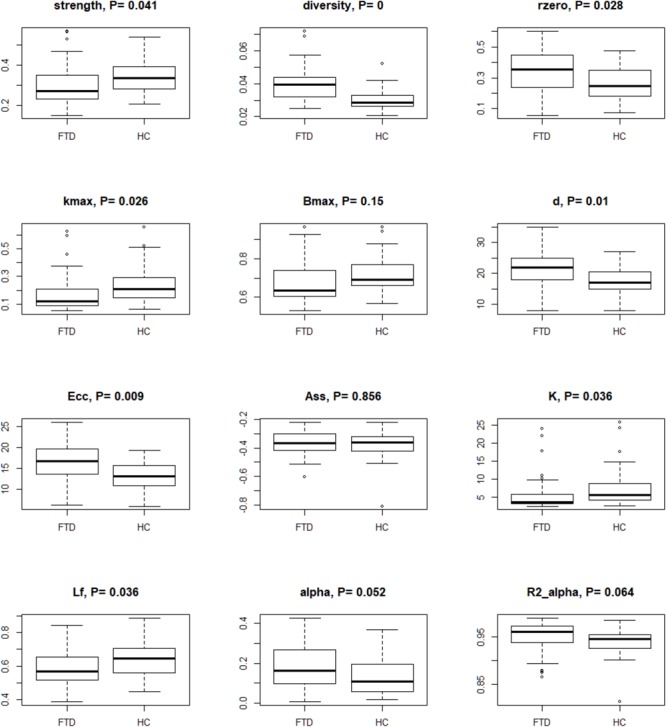
Box-plots and *P*-values of Wilcoxon rank sum test for global connectivity measures and minimum spanning tree global parameters. The null hypothesis of the test is that the distributions of the HC and bvFTD do not differ (i.e., true location shift equal to 0). Data were corrected by age, sex, and age^∗^sex interaction, and *P*-values were adjusted by Benjamini–Hochberg correction. Legend: rzero, zero correlation; kmax, maximum degree; Bmax, maximum betweenness; d, diameter; Ecc, eccentricity; Ass, assortativity; K, degree divergence; Lf, leaf fraction; alpha, extreme value index; R2 alpha, R-square of the extreme value index.

For each subject, we examined the complexity of rs-fMRI data (Saba et al., 2018) using bivariate measures (i.e., strength, diversity, and zero correlation) computed as summary regional values of the wavelet correlations (Supplementary Table 2). Scale 2 was the only reaching significance at these three tests (Figure 2 and Supplementary Table 2). Diversity and zero correlation showed a significant increase in bvFTD respect to HC, while strength showed significant decrease (*P* < 0.05 for every test). The significant increment of wavelet correlation diversity and its percentage of zeros (median HC: 25% vs. median bvFTD: 35%) indicated a decreased heterogeneity and increased null functional connectivity between brain regions in bvFTD compared to HCs. In addition, strength decrease denounces a generalized connectivity weakening in bvFTD respect to HCs.

### Stronger Disorder Limit

By analyzing TOM-based weighted fully connected graphs for each subject (see Materials and Methods), we obtained good fitting for the power function (Figure 2 and Supplementary Table 2). High *R*^*2*^ (R-squared) indices were observed in both groups (0.93–0.97 for HC and 0.90–0.99 for bvFTD). When the extreme value index was estimated ($\hat{\text{α}}$), excluding outliers, low values (0.14–0.57 for HC and 0.10–0.42 for bvFTD) were found. Values of $\hat{\text{α}}$ less than one indicated a strong disorder limit tendency.

### MST Global Graph Metrics

Minimum spanning trees indicated brain connections alterations in bvFTD patients when compared to controls (Figure 2 and Supplementary Table 2). Data suggested a reduction of the degree centrality and leaf fraction, and an increment of distance metrics in the bvFTD group. Their trees were composed by nodes with a lower maximum degree and number of leafs. Moreover, the trees were characterized by a higher inter-distance, translating in a higher diameter and eccentricity in bvFTD compared to HC trees. These measures indicated brain impairments in bvFTD, highlighting less node-connections and loss of efficiency in exchange information capacity, that support a linear-shaped configuration network. Conversely, HC tree metrics showed a better network integration, characterized by parameter values that tend to a star-type configuration (i.e., an increase in the number of hubs and leaf points, and a decrease of diameter and eccentricity). Although assortativity index was not significant, it confirmed the topological hierarchy and biological nature of all MSTs, with assortativity values less than 0, for both groups. Lastly, permutation testing of two-group differences on the average correlation matrices yielded similar results (data not shown).

### MST Topological Two-Group Comparison

**FIGURE 3:**
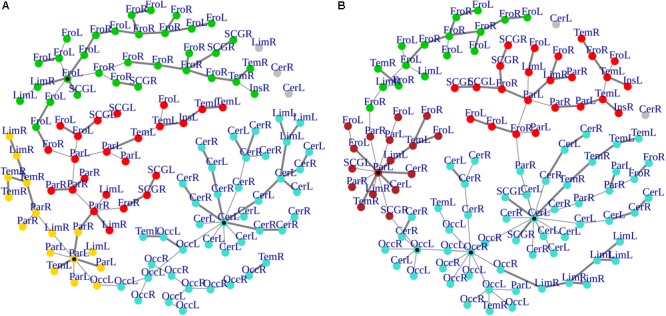
**(A,B)** Clusters and superhighways differences in brain macro-regions. MSTs for both bvFTD and HCs are shown on the left **(A)** and right **(B)** panel, respectively. Clusters are defined by node colors, while thick edges show superhighway paths. Hubs (i.e., nodes having degree centrality >5) are marked with a black dot. Nodes are labeled according to macro-regions (i.e., lobes) membership (see Supplementary Table 1 for label encodings).

Cluster, spatial, and anatomical data were integrated through a network representation, showing the topological properties of the two-group average for HC and bvFTD graphs (see Materials and Methods section). The results of edge partitioning and node clustering are shown in Figure 3A,B, 4A,B. Every node in Figure 3A,B, colored by cluster membership, correspond to a single brain area belonging to a specific macro-region or lobe (i.e., node name), and traversed by two kinds of functional connections: (i) superhighways (bold gray), and (ii) secondary functional routes (thin gray). Finally, Figure 5A,B displays lobe partition (node color), node degree centrality (node size), and superhighways information flow connectivity (edge thickness) for bvFTD and HC groups, respectively.

**FIGURE 4:**
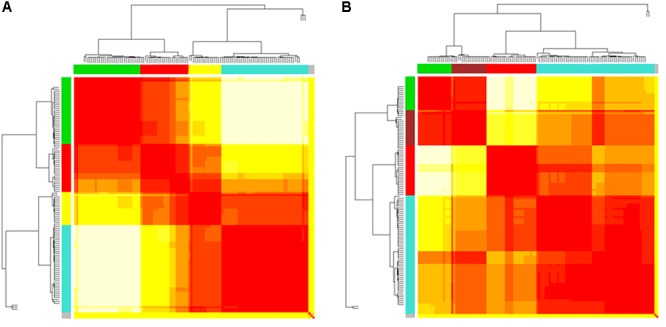
**(A,B)** Heatmaps and dendrograms of brain areas clustering elaboration. Hierarchical subdivision of brain areas and clustering for both bvFTD and HCs are shown on the left **(A)** and right **(B)** panel, respectively. The same number of clusters for both groups is obtained through the application of a cutting-height on dendrograms. We applied a cut-off equal to 0.2 to obtain four clusters (yellow-to-red squares within clustering areas). The four clusters (showed as colored boxes), reveal a clearer subdivision in the bvFTD group **(A)** respect to HCs **(B)**.

**FIGURE 5:**
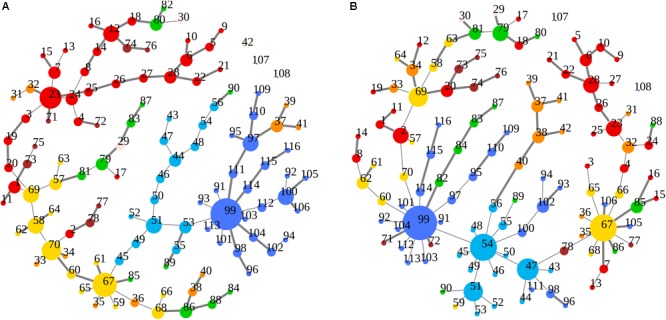
**(A,B)** Node degree centrality and superhighway routes in brain macro-regions. MSTs for both bvFTD and HCs are shown on the left **(A)** and right **(B)** panel, respectively. Macro-regions (i.e., lobes) are defined by node colors. Thick edges show superhighway paths and node size is proportional to degree centrality. Nodes are labeled by AAL 116 region identifier. Identifiers and corresponding areas and macro-regions can be found in Supplementary Table 1. Macro-region color code: Red, Frontal; Green, Temporal; Yellow, Parietal; Orange, Limbic; Cyan, Occipital; Brown, Subcortical Gray Matter; Light pink, Insula; Blue, Cerebellum.

In Figure 3A,B, 5A,B emerged the HC multiple-star structure, whose central nodes, namely Lingual-L, Occipital-Inf-R, Precuneus-L, and Cerebellum-6-L regions (nodes #47, #54, #67, and #99 in Figure 5B), serve as starting points for superhighways and bridge components in the tree backbone. This architecture was impaired in the MST of bvFTD patients, where few conserved stars, namely Precuneus-L, and Cerebellum-6-L (nodes #67 and #99 in Figure 5A), maintained a reduced functional connectivity, leading to a general isolation of Frontal and Temporal areas from central nodes. Network complexity reduction from HC to bvFTD is evident from bvFTD hierarchical clustering in Figure 3A, where Frontal and Temporal areas (green and red clusters, respectively) are almost completely separated from Parietal (yellow cluster) and Occipital-Cerebellar regions (cyan cluster). On the other hand, HC heatmap in Figure 3B, shows two highly connected network communities (green-brown and red-cyan clusters). Specifically, two different groups of Frontal-Parietal regions (brown and red clusters) are highly connected to Frontal (green cluster) and Temporal-Occipital-Cerebellar regions (cyan cluster), respectively. Notably, network star nodes (i.e., Lingual-L, Occipital-Inf-R, Precuneus-L, and Cerebellum-6-L) traverse and integrate these two highly connected communities in HC (Figure 5B). Conversely, bvFTD clusters are much more homogeneous (i.e., areas from the same lobe tend to cluster together), involving fewer and isolated stars. This is evident in Figure 5, where the bvFTD network panel (A) shows a clear lobe segregation (i.e., node color), especially for Frontal (red), Parietal (yellow), Occipital (light blue), and Cerebellum (blue) lobes, while the HC network panel (B) shows a much higher level of integration.

**FIGURE 6:**
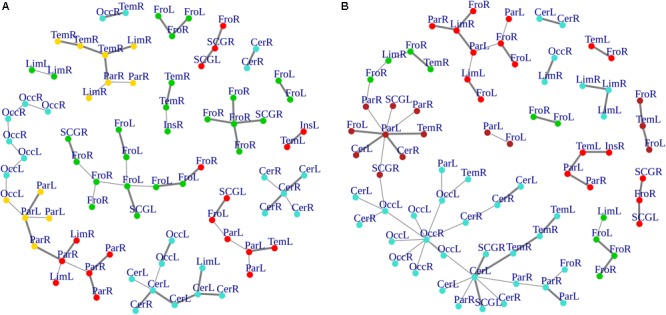
**(A,B)** Graph-edges difference between the two groups. MSTs differences for both bvFTD and HCs are shown. Left **(A)** panel shows bvFTD-HC residual graph, while right **(B)** panel reports the difference HC-bvFTD. Node colors follow cluster membership, as in Figure 3. Nodes are labeled according to macro-regions membership (see Supplementary Table 1 for label encodings).

The survival ratio (i.e., the intersection between graphs calculated as the fraction of links found common in two MSTs) was equal to 42%, defining dissimilar topological structures of MSTs in the two groups. These differences are highlighted in Figure 6, where edges present in the bvFTD graph but not in the HC one panel (A), and vice versa panel (B), are shown. More specifically, highly connected star-clusters seen in the HC group were absent in the bvFTD graph. Conversely, leafs and short linear structures seen in the bvFTD group were absent in the HC graph.

**FIGURE 7:**
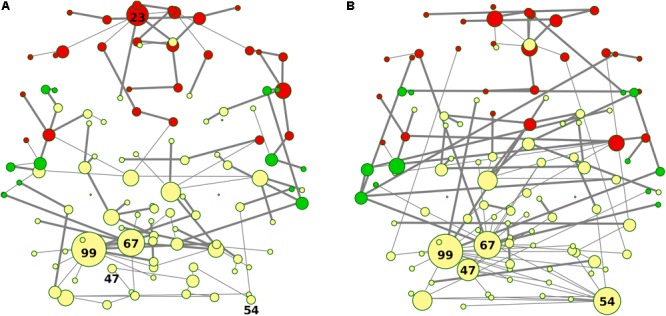
**(A,B)** Spatial location of areas and superhighway routes according to x-y AAL 116 coordinates. MSTs for both bvFTD and HCs are shown on the left **(A)** and right **(B)** panel, respectively. Thick edges show superhighway paths and node size is proportional to degree centrality. Red nodes correspond to Frontal areas, while Temporal areas are colored in green. Hubs (i.e., nodes having degree centrality >5) are labeled by AAL 116 region identifier. Left MST **(A)** reports conserved hubs (areas #67 and #99), lost hubs (areas #47 and #54), and the gained frontal hub (area #23) in bvFTD, respect to HCs.

Nodes and edges in the axial orientation (x-y)-coordinates of the anatomical automatic labeling (AAL 116) brain atlas are shown in Figure 7A,B, providing a complete frontotemporal brain state representation, through the visualization of type, number, and origin of connections, confirming the abnormalities found in bvFTD, compared to HC. The most evident feature is a massive grouping of Frontal areas (red nodes in Figure 7A), disconnected from both Temporal lobes (green nodes in Figure 7A) and other areas (yellow nodes in Figure 7A), including the conserved star nodes Precuneus-L and Cerebellum-6-L (nodes #67 and #99 in Figure 7A). Strikingly, the massive frontal aggregation in bvFTD belongs entirely to a single cluster (red nodes in Figure 5A, 7A), at the center of which is present a new bvFTD-specific star node (Frontal Sup-Medial-L, node #23 in Figure 5A, 7A), indicative of a new isolated functional macro-region in bvFTD. Notably, comparing superhighways distribution between groups (Figure 7A,B), HCs show a deeply intertwined connectivity that integrates star nodes with Frontal and Temporal areas, linking them each other. Conversely, bvFTD superhighway connectivity collapses around conserved hubs and within the Frontal macro-region, causing their isolation.

### Methodological Comparison

Minimum spanning tree network metrics were compared to other classical graph theory approaches (see Materials and Methods), to assess network properties reproducibility and wavelet scale specificity. Network metrics and Wilcoxon rank sum test results are reported in Supplementary Table 2. Beside the MST, only the efficiency/cost trade-off based method (i.e., ECO) showed significant bvFTD/HC specifically at scale 2, while all the other scales were non-informative (i.e., non-significant bvFTD/HC differences). The EBIC-based method reported few significant indices not directly related to information exchange efficiency (i.e., maximum degree, degree divergence, and assortativity), for both scales 1 and 2, indicating poor discriminant power. Lastly, spectral analysis and scale free model-based methods did not show significant case/control differences at any wavelet scale. Notably, both MST and ECO are based on the optimization of the information flow exchange across the functional network, maximizing graph integration and minimizing wiring costs. Common metrics between MST and ECO showing significant results at scale 2 were the same in both methods. However, differently from MST, ECO yielded several disconnected nodes (up to 74) per subject.

## Discussion

In the present study, we evaluated the power of MST representation within the framework of bvFTD, combining rs-fMRI data and graph theory analysis. Different neuroimaging methods have been proposed to highlight brain damage in bvFTD (Whitwell et al., 2011; Rohrer et al., 2015). However, since the description of the BOLD signal (Ogawa et al., 1990), functional neuroimaging allowed to go beyond the mere anatomical description of brain connectivity, identifying functionally connected (i.e., synchronized time-dependent fluctuations of the BOLD signal) networks of cortical and subcortical regions (Seeley et al., 2008). In bvFTD, multiple independent studies identified the frontal brain regions as the affected core networks (Seeley et al., 2009; Zhou et al., 2010; Whitwell et al., 2011; Borroni et al., 2012; Farb et al., 2013; Lee et al., 2014).

Graph theory has already been applied in a few MRI studies on bvFTD, demonstrating disruption of the global topologic organization, increased path length and assortativity, with loss of cortical hubs and network centrality, extending the list of sensible target regions to salience and executive functions (Agosta et al., 2013; Trojsi et al., 2015; Sedeño et al., 2016; Hohenfeld et al., 2018). Despite the wide use of graph theory in the analysis of neurodegenerative disorders, many methodological aspects are left to the experimenter choice, leading to possible confounding results.

When comparing graphs with the same number of nodes (*N*) and edges (*N*-1), the MST is an unambiguous method for brain network analysis, allowing to avoid methodological biases (Tewarie et al., 2015). Although it does not completely replace traditional graph theory approaches, MST remains the simplest and most effective representation of a full graph (Stam et al., 2014), where minimum weight (i.e., maximum connectivity) links constitute significant information flow paths. Moreover, it has been recently demonstrated how MST provides robust network estimates, with results in accordance to classical network analytical data (Otte et al., 2015; Tewarie et al., 2015). As indicated by our methodological comparison (see Results), algorithms based on information flow optimization and wiring cost minimization (including MST and ECO methods) achieved the best bvFTD/HC separation performances (Supplementary Table 2). On the other hand, fixed-correlation threshold and scale free model-based graphs showed poor or non-significant results, indicating that enforcing an arbitrary correlation threshold or network nature (i.e., scale free power law distribution) strongly limit network descriptive power. Conversely, MST has the advantage of requiring no correlation thresholds, network density or *a priori* distribution, ensuring full reproducibility and robustness in different conditions. Furthermore, since our results indicated a strong disorder limit tendency in both groups, the MST was able to preserve the connectivity features of the underlying functional networks (Tewarie et al., 2014). Through our TOM-adjusted edge weights, MSTs and relative parameters conserved their neighborhood node characteristics, highlighting nodes aggregation (i.e., star-type configurations), and providing a valid method to identify a set of shortest paths in MSTs that may be considered as the “critical backbone” of original graph (Tewarie et al., 2014).

Our MSTs suggest that the differences between groups may be attributed to functional alterations of such major organization, since they represent the information-flow highways of the fully connected network. Shape-linear configuration tendency in bvFTD graphs highlights different impairments: high distance between nodes, low centrality parameter values, and a low exchange information capacity (i.e., low network integration). Connection efficiency loss is particularly evident in Figure 7, where the superhighway system in HCs, linking hubs to Frontal and Temporal brain areas, is replaced by a local (i.e., isolated) network surrounding conserved hubs. Functional isolation is a generalized process in bvFTD, where brain areas tend to interact within lobes (i.e., colors in Figure 5), showing a homogeneous brain area distribution, longer distances between hubs, and longer within-lobe superhighways. Therefore, bvFTD functional breakdown is not merely described by connectivity loss, but though disease-specific reorganizations and regularization of the information flow. This contrasts with the marked integration of a healthy functional network, where superhighways serve as shortcuts to connect areas from different brain macro-regions. Network regularization has already been observed as a distinctive FTD trait, respect to other neurodegenerative disorders, including Alzheimer’s disease (de Haan et al., 2009; Zhou et al., 2010). We further investigated this aspect by gathering evidences from both global and local network metrics.

Although global functional parameters (i.e., strength, diversity, and zero correlation) showed a significantly weaker and reduced connectivity in bvFTD, edge-level and node-level features (i.e., superhighways, and node degree and betweenness centrality), highlighted a more complex scenario, explaining some of the key dysfunctions observed in large scale resting-state functional networks, including the DMN, SN, and EN networks (Zhou et al., 2010; Trojsi et al., 2015; Sedeño et al., 2016; Hohenfeld et al., 2018). The first evidence from our data is the formation of a new FTD-specific hub (area #23: Frontal_Sup_Medial_L, Figure 5A), absent in HCs. This hub is the starting point of a huge superhighway, fully extending within frontal lobe, clearly derived from an elongation of the original route in HCs (Figure 5B). Specifically, our data showed the involvement of regions: Frontal_Sup_Medial_L/R, Frontal_Sup_Orb_L/R, Frontal_Mid_Orb_L/R, Frontal_Med_Orb_L/R, Rectus_L/R, and Olfactory_L/R (areas #23, #24, #5, #6, #9, #10, #25, #26, #27, #28, #21, and #22 in Supplementary Table 1 and Figure 5A). Notably, all the elements of this superhighway are part of the DMN (Zhou et al., 2010; Trojsi et al., 2015; Hohenfeld et al., 2018), strongly supporting the evidence of a compensation mechanism and frontal DMN decreased connectivity with areas from other lobes. However, this process is not exclusive of the Frontal lobe. Within-lobe superhighway formation, and consequent isolation, involves also the two conserved nodes in bvFTD: Precuneus_L (area #67, parietal lobe in yellow in Figure 5A) and Cerebellum_6_L (area #99, blue in Figure 5A), where the former plays a key role in the DMN network. Betweenness difference (*b*) is a good local indicator of these impairments at node level (threshold set at *b* = ± 435, (see Supplementary Table 3). Notably, while areas #23 and #67 show a strongly increased node betweenness (*b_23_* = 1545, and *b_67_* = 879) from bvFTD to HCs, area #99 loses a great portion of its centrality (*b_99_* = -1534), supporting the evidence of a functional deterioration of the cerebellar lobule VI, that has been associated with cerebellar atrophy in both bvFTD and Alzheimer’s disease (Guo et al., 2016; Schmahmann, 2016). Nevertheless, area #99 is the center of an enlarged intra-cerebellar superhighway system, showing that superhighway elongation is due to a generalized network centrality reorganization, rather than a region-specific impairment. It has been recently suggested that long-distance connections have an important role in integrating distinct brain areas, leading to a greater functional diversification, robustness, and specialization (Betzel and Bassett, 2018). However, long-range connections number and length is strictly controlled by their metabolic demand. In contrast, bvFTD long within-lobe superhighways seem not to contribute to the overall functional integration, but rather being the result of a compensatory mechanism, in response to a generalized functional deterioration. To verify this hypothesis, we focused our attention to those areas showing a strong betweenness centrality loss (*b* < -435) in bvFTD respect to HCs. The clearest example in our data is given by three connected areas (Figure 5B): Angular_R (area #66, *b_66_* = -1552), Frontal_Sup_R (area #4, *b_4_* = -1359), and Cingulum_Ant_R (area #32, *b_32_* = -1299), being involved in DMN, EN, and EN/SN, respectively. As shown in Figure 5B, these three areas connect the Precuneus_L (parietal area #67) and its superhighway system to the frontal superhighway starting from area #23 (Frontal_Sup_Medial_L), that in bvFTD is markedly enlarged (Figure 5A). In the bvFTD network, area #23 is a new hub, and the #23–67 connection is now a much longer and linear path, suggesting that superhighway elongation could be a compensatory reaction to a less efficient network integration. Notably, Cingulum_Ant_R (area #32) is part of the limbic system, involved in both DMN and salience/executive functions, suggesting that limbic system failure could be an underlying cause of the global network rearrangement observed in bvFTD subjects.

A further support to this hypothesis is represented by the disruption of two HC network hubs (Figure 5B): Occipital_Inf_R (area #54, *b_54_* = -4054) and Lingual_L (area #47, *b_47_* = -2913), replaced by a long linear-shaped sequence of occipital areas (Figure 5A), denouncing a massive loss of degree and betweenness centrality also in this part of the network. Beside hubs, two adjacent connector areas experienced huge loss of betweenness: Fusiform_R (area #56, *b_56_* = -531) and ParaHippocampal_R (area #40, *b_40_* = -540). Strikingly, while the former is a direct bridge to the hub group, the latter is the first node of a limbic superhighway (Figure 5B), whose nodes occupy distinct peripheral positions in bvFTD (Figure 5A), including: Hippocampus_L/R (area #37–38), ParaHippocampal_L/R (areas #39–40), and Amygdala_L/R (areas #41–42).

Collectively, these evidences show an underlying involvement of the limbic system in the observed bvFTD functional deterioration, associated to the well-studied impairments affecting emotion recognition, social inference, and executive functions typical of this neurodegenerative disorder (Zhou et al., 2010; Trojsi et al., 2015; Sedeño et al., 2016; Hohenfeld et al., 2018).

## Conclusion

The present work had the primary goal of highlighting alterations in brain connectivity of bvFTD subjects, providing at the same time a detailed description of the observed functional impairments, and insights about their possible causes. According to the most recent fMRI literature (Guo et al., 2017; Cui et al., 2018), we applied an MST model to wavelet correlation matrices from bvFTD and HC subjects, exploiting three strongpoints of MST-based methods: (i) assumption-free network construction and reproducibility, (ii) independence from node and edge number during network comparison, and (iii) simplicity of representation (i.e., three branching is directly interpretable in terms of most efficient shortest paths). On the other hand, MSTs have one main limitation: the resulting network is a sparse representation, implicitly excluding triangular connections, thus causing non-applicability of some common clustering metrics (e.g., transitivity and coreness) and evaluation of the network small-worldness. However, we coped with this issue by using TOM (Zhang and Horvath, 2005) and the extreme value index evaluation (Van Mieghem and Magdalena, 2005).

The combination of this theoretical model with rs-fMRI data allowed us not only to generate a clear picture of the functional divergence of bvFTD from HCs, but also to shed light on the possible causes of topological and functional rearrangements, and compensatory mechanisms, underlying cognitive, social, and executive impairments characterizing bvFTD phenotype.

Further developments to the present work, that now constitute main limitations, are represented by: (i) the lack of clinical and/or metabolic parameters that could confirm or reveal new causal hypotheses, and (ii) a combination with anatomical variables (e.g., gray matter mass), to achieve a better model resolution, and associate degenerative processes to functional deterioration.

Collectively, the application of MST-based analysis to rs-fMRI data looks a promising way to clarify the role of degenerative processes involved in FTD functional breakdown, improving the discovery of new fMRI biomarkers.

## Data Availability

Pre-processed and parceled data (ItalianFTD-fMRI_May2018) for this study can be found in the SOURCEFORGE repository at: https://sourceforge.net/projects/bionet-finder/.

## Author Contributions

VS performed the literature research, drafted the manuscript, performed the data analysis and R coding, and wrote, reviewed, and edited the manuscript. EP conceived and designed the study, acquired the data, analyzed the image, and wrote, reviewed, and edited the manuscript. VC performed the literature research and analyzed the image. FP revised the data analysis, results, and discussion, and wrote, reviewed, and edited the manuscript. SG, OZ, RG, and AP acquired the data. BB conceived and designed the study, recruited the patients, drafted the manuscript, and supervised the study. MG conceived and designed the study, administered the project, performed the data analysis and R coding, and wrote, reviewed, and edited the manuscript. All authors have read and approved the final manuscript.

## Conflict of Interest Statement

The authors declare that the research was conducted in the absence of any commercial or financial relationships that could be construed as a potential conflict of interest.

## Footnotes

1 http://marsbar.sourceforge.net/

## Abbreviations

AAL: automated anatomical labeling
bvFTD: behavioral variant Frontotemporal Dementia
HC: healthy controls
MST: minimum spanning tree
ROI: regions of interest
rs-fMRI: resting state functional MRI
SPT: shortest path tree
TOM: topological overlap measures
